# Can Platelet Count and Mean Platelet Volume be Used as Markers of Postdural Puncture Headache in Obstetric Patients?

**DOI:** 10.1155/2020/6015309

**Published:** 2020-08-07

**Authors:** Osman Uzundere, Cem Kıvılcım Kaçar, Sedat Kaya

**Affiliations:** TR HSU Diyarbakır Gazi Yaşargil TRH, Department of Anesthesiology and Reanimation, Elazığ Yolu 10. Km Üçkuyular Mevkii 21070, Diyarbakır, Turkey

## Abstract

**Objective:**

In this study, considering the importance of platelet function in inflammatory processes, we explored whether there are relationships of platelet indices with postdural puncture headache (PDPH) and pain developing after use of spinal needles and whether patient characteristics contribute to the development of PDPH.

**Methods:**

This prospective, observational study included 76 patients (Group 1) with PDPH and 93 patients (Group 2) without PDPH. The postoperative hemoglobin, hematocrit, platelet count (PC), and mean platelet volume (MPV) values were recorded, along with age, blood type, Rh factor, gravida, parity, and gestational age. In addition, the time of the onset of pain was recorded in patients who complained of a postspinal headache.

**Results:**

Hemoglobin and hematocrit values in Group 1 were significantly lower than in Group 2 (both, *p*=0.024). The PC of Group 1 was significantly higher than that of Group 2 (*p* < 0.001), whereas the MPV was significantly lower (*p* < 0.001). The area under the curve (AUC) values were significant for hemoglobin, hematocrit, PC, and MPV (*p*=0.022, *p*=0.024, *p* < 0.001, and *p* < 0.001, resp.). For MPV, the AUC value was 0.293, sensitivity was 1%, and specificity was 99%. The highest likelihood ratio (LR+) value was 1.22 at a cut-off value of 13.3 fL. For the PC, the AUC value was 0.666, the sensitivity was 9%, and the specificity was 99%, while the highest LR + value was 8.56 at a cut-off value of 352 × 10^9^/L. There was no significant relationship between the parameters examined and the onset of pain.

**Conclusion:**

In this study, the PC was higher and MPV was lower in obstetric patients with PDPH compared with the control group. However, we also found that these two values cannot be used as markers of PDPH.

## 1. Introduction

There has been a significant increase in cesarean delivery rates in many countries [1]; thus, appropriate anesthesia during the procedure has become more important. Anesthesia techniques for cesarean section include general and regional anesthesia, such as spinal, epidural, and combined spinal-epidural anesthesia [2].

Obstetric patients may experience serious complications from regional anesthesia, which can lead to morbidity or mortality. One of the most important potential complications is postdural puncture headache (PDPH) [3]. The incidence rate of PDPH after spinal anesthesia is approximately 0.5–2%, while it reaches 45–80% with epidural anesthesia [4–7]. PDPH, which develops after cesarean section, significantly restricts the physical activity of the mother and increases hospital costs by increasing the length of stay [8].

Known risk factors for PDPH include young age, female gender, pregnancy, vaginal delivery, previous history of PDPH, history of chronic headache (such as migraine), and low body mass index, [5, 9–12]; risk factors arising from the procedure itself include the type and diameter of the needle, shape of the dural fibers after cutting by the spinal needle, number of interventions, and amount of cerebrospinal fluid (CSF) lost during the procedure [5, 6, 9, 13–17].

Although many mechanisms have been proposed to explain the pathophysiology of postdural headache, PDPH is thought to develop mainly due to two main factors, low cerebral fluid volume (intracranial hypotension) due to CSF leakage or stretching of pain-sensitive structures [4, 5, 9, 18]. Furthermore, in a recent cadaver study, electromicroscopic examination of lesions formed after spinal puncture performed with different types (and angles) of needles showed that spinal needles cause traumatic lesions on the dura and arachnoid membranes [18]. In another study, a patient with postspinal headache who did not respond to epidural blood patch treatment, which is commonly used for PDPH, was treated with platelet-rich plasma as an alternative [19]. The epidural patching with platelet-rich plasma was not effective in preventing mechanical CSF leakage. Nevertheless, platelets play an important role in regeneration and healing through the inflammatory pathway in this area [19].

The platelet count (PC) and functions are very important for hemostasis in normal physiological and pathological processes. At the same time, changes in PC and size occur during inflammatory processes [20]. It has been shown in several studies that there are changes in platelet indices in many cases, such as severe infection, trauma, systemic inflammatory response syndrome, liver diseases (such as cirrhosis), and thrombotic diseases [21, 22]. In addition, platelet indices have been reported to have a diagnostic value in inflammatory diseases such as inflammatory bowel diseases, rheumatoid arthritis, ankylosing spondylitis, ulcerative colitis, and atherosclerosis [23]. Previous studies have shown that mean platelet volume (MPV), as a platelet parameter, can be used to diagnose various diseases [20, 23–27]. An increased MPV is suggestive of larger and more active platelets in the circulation; these active platelets have higher intracellular granule content and greater adhesion capabilities [20].

In our study, the primary endpoint was to investigate relationship between the development of PDPH and platelet indices which are PC and MPV. Whether the characteristics of patients had an effect on the time of development of PDPH was assessed as a secondary endpoint.

## 2. Methods

### 2.1. Study Design, Population, and Data

This study was performed at Diyarbakir Gazi Yaşargil Training and Research Hospital between March 2018 and September 2019, following ethics committee approval (02/16/2018). All patients who participated in this prospective observational study were informed about the procedures involved and provided informed consent. The study was conducted in accordance with the 2008 Helsinki Declaration.

Patients who had no additional disease, underwent elective caesarean section under spinal anesthesia, had an American Society of Anesthesiologists (ASA) grades I–II, and were admitted to the anesthesia outpatient clinic during the postoperative period were included in the study. The control group was randomly selected from the patients who underwent elective cesarean delivery under spinal anesthesia during the study period and at the same time did not have any additional disease or headaches in the postoperative period. A 27-G pencil-point spinal needle (Egemen, TMT Biomedical Equips. Ind. Trade, Co., Ltd., İzmir, Turkey) and a guide needle were used for spinal anesthesia in all patients. All patients included in the study underwent spinal anesthesia using the same method by two experienced physicians.

**Figure 1:**
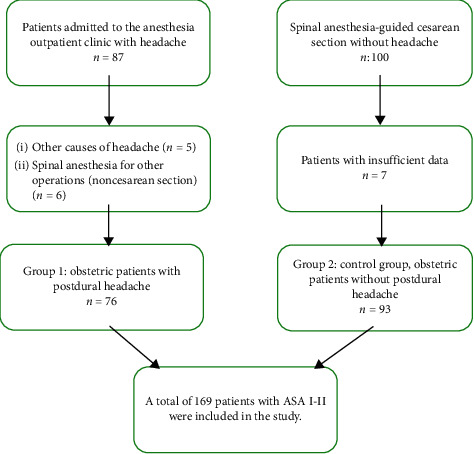
Study flow diagram.

Patients operated on for any reason other than cesarean section (for gynecological reasons) and those who underwent emergency cesarean section (for fetal distress or preeclampsia), had a history of bleeding disorder (thrombocytopenia or coagulation factor deficiency), had a history of PDPH, had a chronic disease (e.g., lung, heart, liver, or inflammatory disease), had a history of regular drug use, were of ASA grades III–IV, or were admitted for a headache other than PDPH were excluded from the study. In addition, patients who underwent spinal anesthesia with needles other than 27-G pencil-point and guide needles were excluded from the study. After applying the exclusion criteria, 76 patients with PDPH (Group 1) and 93 patients without PDPH (Group 2) were finally included in the study (Figure 1).

A diagnosis of PDPH was made in patients who presented to the anesthesia outpatient clinic with a headache, according to the diagnostic criteria of The International Classification of Headache Disorders, 3rd edition (ICHD-3), published by The International Headache Society (IHS) in 2018 [28].

The postoperative hemoglobin, hematocrit, PC, and MPV values of all patients included in the study were recorded along with age, blood type, Rh factor, gravida, parity, and gestational age. In addition, the time of pain onset was recorded in patients who presented with PDPH.

### 2.2. Laboratory Tests

The MPV and other laboratory values were determined by analysis of blood samples obtained from patients who presented to the anesthesia outpatient clinic with PDPH after examination. The complete blood counts of the patients in the control group were obtained during the postoperative period using the BC-6800 instrument (Shenzhen Mindray Bio-Medical Electronics Co., Shenzhen, China). According to the standards of our laboratory, MPV values of 6.5–12.0 fL were considered normal.

### 2.3. Statistical Analysis

G-Power software (version 3.1.9.4; University of Kiel, Kiel, Germany) was used to calculate the required sample size based on a previous study [29]. The minimum number of patients required was 132 (56 in the experimental group and 76 in the control group), assuming a two-tailed alpha error of 0.05, power of 0.80, allocation ratio of *N*2/*N*1 = 1.36, and effect size of 0.5.

SPSS 16.0 software for Windows (SPSS Inc., Chicago, IL, USA) was used for the statistical analysis. Continuous data are expressed as mean and standard deviation; categorical data are expressed as frequency and percentage. The categorical data of the groups were compared using the chi-square test. The Kolmogorov-Smirnov test was used to determine whether the numerical data were normally distributed. Student's *t*-test was used to analyze data with a normal distribution, while the Mann–Whitney *U* test and Kruskal Wallis test were used to analyze nonnormally distributed data. The area under the curve (AUC) and sensitivity-specificity values for hemoglobin, hematocrit, PC, and MPV were calculated using receiver operating characteristic (ROC) analysis. The likelihood ratio (LR+) was used to determine optimal cut-off values. In all comparisons, *p* < 0.05 was considered significant.

## 3. Results

During the study period, there were 23,000 deliveries in our hospital, 6,487 of which were by cesarean section. Spinal anesthesia was applied in 93.88% (6,089 patients) of patients who underwent a cesarean section. The rate of PDPH development in this patient group was 1.24% (76/6,089). The mean age of the 169 patients included in the study was 29.8 ± 6.28 years, the mean gravida was 2.88 ± 1.33, the mean parity was 2.75 ± 1.18, and the mean gestational age was 37.9 ± 0.99 years. The mean time to onset of pain was 2.71 ± 1.17 days in patients with PDPH admitted to the anesthesia outpatient clinic.

Table 1 compares the groups in terms of age, gravida, parity, gestational age, hemoglobin, hematocrit, PC, and MPV. The hemoglobin and hematocrit values of the PDPH group were significantly lower than those of the control group (both, *p*=0.024). When the groups were compared in terms of platelet indices, the PC values of the PDPH group were significantly higher than those of the control group (*p* < 0.001), whereas the MPV value was significantly lower than that of the control group (*p* < 0.001) (Table 1).

**Figure 2:**
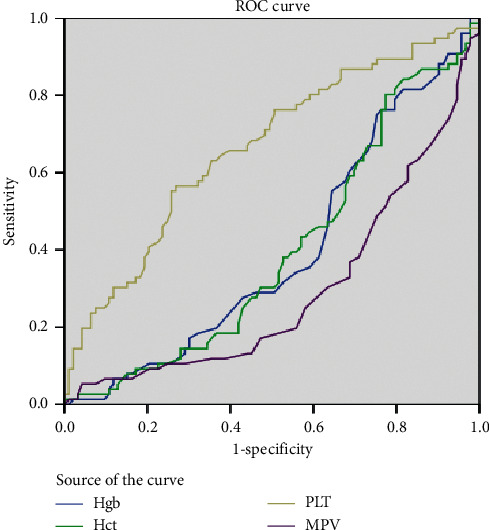
ROC curves for hemoglobin, hematocrit, platelet and main platelet volume. AUC_Hgb_ = 0,398; AUC_Hct_ = 0,399; AUC_PC_ = 0,664; AUC_MPV_: 0,293. Hgb: Hemoglobin; Hct: Hematocrit; PC: Platelet count; MPV: Main platelet volume.

The AUC values, calculated by ROC analysis, were significant for hemoglobin, hematocrit, PC, and MPV (*p*=0.022, *p*=0.024*p* < 0.001 and *p* < 0.001, resp.) (Figure 2). The AUC value for MPV was 0.293; sensitivity was 1% and specificity was 99%, and the highest LR+ value was 1.22, at a cut-off value of 13.3. The AUC value for PC was 0.6664; sensitivity was 9% and specificity was 99%, and the highest LR+ value was 8.56, at a cut-off value of 352. The sensitivity and specificity values corresponding to the highest LR+ for hemoglobin and hematocrit are shown in Table 2.

In 76 patients who developed PDPH, the correlations of onset of postdural headache with age, gravida, parity, gestational age, hemoglobin, hematocrit, PC, and MPV values were calculated; the results are shown in Table 3. No significant relationship was found between any of the parameters examined and the time of pain onset.

## 4. Discussion

In this study, PC values, as one of the platelet indices, were higher and MPV values were lower in patients with PDPH compared with patients without PDPH. On electromicroscopic evaluation, the dura mater is characterized as vascularized connective tissue, with a thickness of around 400 *μ*m, composed of fibers randomly distributed around 80 concentric layers (known as dural lamina), while the arachnoid layer has a thickness of around 40 *μ*m [30, 31]. The arachnoid membrane, the main function of which is to act as a barrier, may limit CSF leakage from the traumatized hole after regional anesthesia [31]. Therefore, the amount of CSF lost from the traumatized hole, and the rate of closure of the arachnoid lesion, may be important with regard to PDPH onset and persistence. Reina et al. stressed a possible role of the arachnoid layer, which lacks the elastic properties of the dural layer, in the closure of lesions in the dural and arachnoid membranes and also found that the primary damage leading to PDPH development is in the form of damage to the arachnoid membrane [18].

In addition to leukocytes, platelets also play an active role in the damage and healing processes of dural and arachnoid tissues associated with spinal needles and inflammation in traumatized tissue. During inflammation, while the PC increases, active, large platelets containing procoagulants and proinflammatory cytokines are observed in the circulation [20]. Hemostasis begins in minutes after tissue damage, causing platelets to accumulate in the damaged area. Platelets accumulating in this region contain platelet-derived growth factor (PDGF), epidermal growth factor (EGF), and fibroblast growth factor (FGF), all of which accelerate wound healing, as well as cytokines (which play a role in hemostasis) [32]. Cytokines also play a role in wound healing, by increasing collagen synthesis and tissue granulation [33]. Platelets can be activated by various stimuli [34]. Due to these properties, inflammation, immune cell activation, tissue regeneration, and other physiological and pathological processes are closely related to hemostasis. These responses are thought to arise through the activation of receptor-signaling pathways specific to certain stimuli [35].

Although no report directly supports a relationship between headache and platelet indices after dural puncture, epidural blood patch and platelet-rich plasma-based methods involve platelets [19, 36, 37]; the healing properties of platelets may have contributed to the positive responses to these procedures obtained when applied in PDPH cases. Dodge et al. evaluated the relationship between smoking and PDPH and found that PDPH was less common in their smoker group. Although the mechanism remains unclear, they suggested that smoking promotes blood clotting and facilitates dural closure [38]. Accordingly, we hypothesize that there is a relationship between platelet indices and PDPH.

MPV, which reflects platelet size and production rate in bone marrow, is a commonly used parameter to evaluate platelet activity and function [20, 39–41]. MPV has been studied in the context of many pathological conditions, including cardiovascular, respiratory, inflammatory, and neoplastic diseases, and has been reported to be an important marker in some of these diseases [24–27, 42–46]. The increase in platelet aggregation seen during normal pregnancy is reflected in the increase in platelet synthesis and MPV [29]. Studies on MPV in obstetric patients mostly included preeclamptic patients, and it has been reported that MPV values are high in severely preeclamptic patients [25, 29, 42, 47]. In our study, although the PC levels were high in patients with PDPH, the low MPV levels may indicate an insufficient active platelet response in these patients; this suggests that MPV plays an important role in the development of PDPH.

In our study, although the MPV value was found to be significantly different between the PDPH patients and controls, MPV may not be an appropriate marker for PDPH because of its low AUC, LR+, and sensitivity values. Similarly, PC is not suitable for detecting PDPH because of its low sensitivity (although its AUC and LR+ values are higher than those of MPV). In a recent study by Tapar [33], there was no significant difference between patients with PDPH and controls in terms of MPV, except postoperatively; MPV values were significantly higher postoperatively in the PDPH group. Thus, MPV may have utility for predicting PDPH in some circumstances.

In our study, the incidence rate of PDPH was 1.24%, consistent with the incidence of PDPH due to spinal anesthesia in pregnant women reported in the literature [4, 5, 48].

We found no differences between the PDPH and control groups in age, gravida, parity, or gestational age when examining the risk factors for PDPH development. This may be because our population consisted of obstetric patients drawn from a particular age group, where this group was identified as an at-risk group for PDPH in previous studies [5, 9–11]. Studies with different patient groups may yield different results.

In our study, hemoglobin and hematocrit values in the PDPH group were lower than in the control group. However, similar to other studies, these values did not show sufficient sensitivity or specificity for use as markers of PDPH. We believe that the significant differences between the groups may be related to the higher degree of localized damage due to spinal anesthesia in the PDPH patients compared to the controls. Sargin et al. found that postoperative hemoglobin and hematocrit values in patients with PDPH were not significantly higher than in the control group [47]. In the other study on this topic, postoperative hemoglobin and hematocrit values in the PDPH group were low, although not significantly different compared to those of the control group [33]. Further studies are required to fully explore this topic. Although varying PDPH onset times have been reported in the literature [6, 8, 9], 90% of PDPHs occur within the first three days after intervention. In rare cases, PDPH onset has been reported between five and fourteen days. Our results were largely consistent with the results of previous studies.

Finally, we found that age, gravida, parity, gestational age, hemoglobin, hematocrit, PC, and MPV were not associated with the time of pain onset. Further studies are required exploring the factors associated with time of pain onset.

## 5. Conclusion

In conclusion, we found that (1) obstetric patients who developed PDPH had higher PC and lower MPV values compared with the control group, (2) hemoglobin, hematocrit, PC, and MPV cannot be used as markers of PDPH, (3) there was no difference between obstetrical patients with PDPH and the control group in age, gravida, parity, or gestational age, and (4) age, gravida, parity, gestational age, hemoglobin, hematocrit, PC, and MPV were not associated with the time of pain onset. Although there was a change in PC and MPV in PDPH patients, they cannot be used as markers of PDPH.

### 5.1. Limitations

There were some limitations to our study. First, it used a single-center design. Therefore, our results may not generalize to other communities and countries. Second, only obstetric patients were included in the study; including other patient groups (such as orthopedic, urological, and general surgery cases) could reveal additional risk factors for PDPH.

## Figures and Tables

**Table 1:** Basic characteristics of the patients and laboratory values (Mean ± SD).

	Group 1 (*n* = 76)	Group 2 (*n* = 93)	*p* value

Age (years)	30,00 ± 6,62	29,64 ± 6,02	0,716^*∗*^
Gravidity (times)	2,96 ± 1,45	2,82 ± 1,23	0,819^#^
Parity (times)	2,78 ± 1,28	2,73 ± 1,09	0,914^#^
Gestational week (week)	38,00 ± 1,08	37,94 ± 0,92	0,702^#^
Hgb (mg/dl)	10,85 ± 1,32	11,37 ± 1,58	0,024^*∗*^
Hct (%)	33,98 ± 7,44	34,79 ± 4,28	0,024^#^
Platelet count (×10^9^/L)	256,940 ± 81,400	215,000 ± 61,200	<0,001^#^
Mean platelet volume (fL)	9,84 ± 1,23	10,66 ± 1,12	<0,001^*∗*^
The time of the beginning PDPH^&^ (day)	2,71 ± 1,17	—	—

^*∗*^Student *t*-test; ^#^Mann–Whitney *U* test; ^&^Postdural puncture headache.

**Table 2:** The results of ROC^*∗*^ analysis for hemoglobin, hematocrit, PC^*∗∗*^ and MPV^*∗∗∗*^.

Parameter	AUC^#^	SE^##^	*p* value	95% confidence interval		LR+	Cut-off value	Sensitivity (%)	Specificity (%)
				Lower bound	Upper bound				

Hemoglobin	0,398	0,044	0,022	0,312	0,483	1,02	8,05	100	3
Hemotocrit	0,399	0,044	0,024	0,313	0,484	1,22	44,2	1	99
PC	0,664	0,042	<0,001	0,582	0,747	8,56	352,000	9	99
MPV	0,293	0,041	<0,001	0,213	0,372	1,22	13,3	1	99

^*∗*^ROC: receiver operating characteristic; ^*∗∗*^PC: Platelet count; ^*∗∗∗*^MPV: Main platelet volume; ^#^AUC: Area under curve; ^##^SE: Standard error; LR+: Likelihood ratio.

**Table 3:** Correlation between the onset of the pain and age, gravidity, parity, gestational week, hemoglobin, hematocrit, PC^*∗*^ and MPV^#^ in patients with PDPH^&^.

	The time of the beginning PDPH			
	Pearson's correlation	Spearman's correlation	*p* value	*n*

Age	0,046	—	0,692	76
Gravidity	—	0,103	0,377	76
Parity	—	0,088	0,447	76
Gestational week	—	0,015	0,895	76
Hemoglobin	−0,012	—	0,921	76
Hematocrit	—	0,019	0,872	76
PC	—	0,149	0,199	76
MPV	−0,201	—	0,082	76

^*∗*^Platelet count; ^#^Main platelet volume; ^&^Postdural puncture headache.

## Data Availability

The data used in this article was collected through the hospital computer system of Diyarbakır Gazi Yaşargil Training and Research Hospital. The data used to support the findings of this study are available from the corresponding author upon request.
